# Sustainable planning in a malaria vector control program: a study in Pesawaran, Indonesia

**DOI:** 10.1186/1475-2875-11-S1-P129

**Published:** 2012-10-15

**Authors:** Dewi Susanna, Kholis Ernawati, Umar F Achmadi

**Affiliations:** 1 Department of Environmental Health, Faculty of Public Health, Universitas Indonesia, Kampus Ul Depok, Indonesia 16424; 2 Centre for Environment, University of Indonesia, Indonesia

## Background

One effective way to control malaria is through vector control [1,2]. Pesawaran is a malaria endemic area with a number of Annual Parasite Incidence (API) of 2.97. [3] The formulation of vector control programs in endemic areas should consider the principles of sustainability [4]. The purpose of this study is to formulate the concept of the development of malaria vector control programs in Pesawaran considering sustainability principles.

## Method

The study was conducted in the area of Pesawaran, Indonesia which has malaria receptive areas along the coast [4]. Data was obtained from a study of literature, in-depth interviews and questionnaires to experts. The experiment was conducted in April-June 2012. The analysis method used is descriptive qualitative as well as quantitative and Analytic Hierarchy Process (AHP). AHP can be used to solve problems related to tangible and intangible factors. Data, ideas, and intuition can be set by using a logical hierarchy structure. Hierarchy is the arrangement of factors/elements of the existing problems that can be set/controlled [5]. Data processing fees expert AHP using software version 11.

## Results

District Pesawaran has endemic malaria receptive areas. Approximately 68.0% of the total patients in health centers Hanura Malaria, 16.9% were in health centers kike and the rest, 15.1% were in health centers Padang Mirror. There were high numbers of cases of malaria in both these areas, because of the many mosquito breeding places such as abandoned farms [4]. Hierarchical model alternative malaria vector control programs as recommended by WHO and in accordance with the conditions of the research area is the management of abandoned farms from becoming mosquito breeding places, chemical and biological larvacide, Insecticide outdoor residual spraying and insecticide Indoor residual spraying (IRS indoor and outdoor) [2]. Based on a literature study aspects into consideration in the selection of an alternative is the social, economic, environmental, technological, and institutional [2,4,6]. Data processing by expert software choice v. 11 shows that the best course of malaria vector control that is able to maintain the quality of the environment in Pesawaran is turned off so as not to be neglected pond breeding places of mosquitoes (62%), chemical and biological larvacida (23%), and the IRS indoor and outdoor (15%). The order of aspects to be considered in the selection of alternative mosquito control is the social aspect (0.312), the environment (0.258), economic (0.201), technology (0.131) and institutional (0.97). The social aspect (sub criteria: community participation, involvement of other stakeholders, employment, and minimal conflicts in society) tops the list to be considered in the selection of malaria vector control program. Cooperation stakeholder and public participation to determine the success of vector control in an endemic area [4,6].

## Conclusion

Priority malaria vector control in endemic areas Pesawaran, considering the principle of sustainability, namely environmental management on farms neglected in order not to become breeding places and the biggest aspects to be considered a priority selection is the social aspect.
